# Gestational Age and Neonatal Brain Microstructure in Term Born Infants: A Birth Cohort Study

**DOI:** 10.1371/journal.pone.0115229

**Published:** 2014-12-23

**Authors:** Birit F. P. Broekman, Changqing Wang, Yue Li, Anne Rifkin-Graboi, Seang Mei Saw, Yap-Seng Chong, Kenneth Kwek, Peter D. Gluckman, Marielle V. Fortier, Michael J. Meaney, Anqi Qiu, for the GUSTO Study Group

**Affiliations:** 1 Department of Psychological Medicine, Yong Loo Lin School of Medicine, National University of Singapore, National University Health System, Singapore, Singapore; 2 Department of Biomedical Engineering, National University of Singapore, Singapore, Singapore; 3 Singapore Institute for Clinical Sciences, the Agency for Science, Technology and Research, Singapore, Singapore; 4 Saw Swee Hock School of Public Health, National University of Singapore, Singapore, Singapore; 5 Department of Obstetrics & Gynaecology, Yong Loo Lin School of Medicine, National University of Singapore, National University Health System, Singapore, Singapore; 6 Department of Maternal Fetal Medicine, KK Women’s and Children’s Hospital, Singapore, Singapore; 7 Liggins Institute, University of Auckland, Auckland, New Zealand; 8 Department of Diagnostic and Interventional Imaging, KK Women’s and Children’s Hospital, Singapore, Singapore; 9 Departments of Psychiatry and Neurology & Neurosurgery, McGill University, Montreal, Canada; 10 Clinical Imaging Research Centre, National University of Singapore, Singapore, Singapore; Harvard Medical School, United States of America

## Abstract

**Objective:**

Understanding healthy brain development *in utero* is crucial in order to detect abnormal developmental trajectories due to developmental disorders. However, in most studies neuroimaging was done after a significant postnatal period, and in those studies that performed neuroimaging on fetuses, the quality of data has been affected due to complications of scanning during pregnancy. To understand healthy brain development between 37–41 weeks of gestational age, our study assessed the *in utero* growth of the brain in healthy term born babies with DTI scanning soon after birth.

**Methods:**

A cohort of 93 infants recruited from maternity hospitals in Singapore underwent diffusion tensor imaging between 5 to 17 days after birth. We did a cross-sectional examination of white matter microstructure of the brain among healthy term infants as a function of gestational age via voxel-based analysis on fractional anisotropy.

**Results:**

Greater gestational age at birth in term infants was associated with larger fractional anisotropy values in early developing brain regions, when corrected for age at scan. Specifically, it was associated with a cluster located at the corpus callosum (corrected p<0.001), as well as another cluster spanning areas of the anterior corona radiata, anterior limb of internal capsule, and external capsule (corrected p<0.001).

**Conclusions:**

Our findings show variation in brain maturation associated with gestational age amongst ‘term’ infants, with increased brain maturation when born with a relatively higher gestational age in comparison to those infants born with a relatively younger gestational age. Future studies should explore if these differences in brain maturation between 37 and 41 weeks of gestational age will persist over time due to development outside the womb.

## Introduction

Understanding healthy fetal brain development is essential to be able to detect abnormal developmental trajectories due to developmental disorders [1]. Previous studies showed that younger gestational age at birth has been associated with reduced maturation of the brain. However, current knowledge derives largely from studies with premature infants [2]–[8], which do not inform us about healthy brain development in term born infants. Second, different methods of scanning have been used. Most of the existing literature relies upon ultrasound or traditional structural magnetic resonance imaging (MRI). However, Ment et al. (2009) pointed out in a review article that abnormal neurodevelopmental trajectories associate with microstructural abnormalities in the brains, especially white matter abnormalities such as diffuse injury of white matter with neuronal and axonal disruption [9]. Nevertheless, microstructure development is best measured by diffusion tensor imaging (DTI) [10], with white matter maturation characterized by increasing fractional anisotropy (FA) and decreasing mean diffusivity (MD) [11]. Third, neuroimaging is often done after a significant postnatal period. The majority of existing studies with term infants used case-control designs and compared term infants to late preterm infants (34–37 weeks), preterm (32–34 weeks) and/or very preterm infants (gestational age at birth <32 weeks) [2]–[6], scanned at term-equivalent age, after a significant postnatal period. However, the days from birth to imaging can account for potential postnatal brain growth with many ex-uterine environmental factors will influence neuronal connectivity [12]. Indeed, a recent study showed the extraordinary rates of structural growth in the very early postnatal period [13]. As such imaging after a significant postnatal period will not inform us much about the *in utero* brain development and it remains unclear if altered neuroconnectivitity emerges prenatally [14]. Hence, performing DTIs during the third trimester directly on fetuses would be most ideal. Recently some studies used neuroimaging during pregnancy to understand fetal brain development [1], [12]–[15]. However, the understanding of maturation of fetal neural networks remains limited due to many complications of fetal scanning during pregnancy such as difficulties to recruit healthy pregnant women for DTI scans, the size of mothers during third trimester, artifacts due to movements of the fetus and breathing of the mother, and subsequently the long scanning duration (which is especially challenging for women in the third trimester) [14], [16]. Hence, although during the third trimester growth and neurogenesis of the fetal brain increase significantly, the direct effects of a relatively younger gestational age at birth in healthy term infants on these processes are not yet well understood [14], [17].

To understand the establishment of brain connections within healthy term born infants, imaging soon after birth is essential to reduce the effects of postnatal brain maturation. To the best of our knowledge there have been no DTI studies to date that have examined differences brain maturation shortly after being born in healthy term infants. The aim of this study is to investigate the differences in *in utero* brain maturation between 37 and 41 weeks of gestational age in a large cohort of healthy term infants in Singapore. We hypothesize that among healthy term born infants, a younger gestational age at birth is associated with reduced white matter maturity in the brain in comparison to healthy term born infants born with an older gestational age at birth.

## Methods

### Subjects

One hundred eighty nine infants who participated in a birth cohort study, Growing Up in Singapore Towards Healthy Outcomes (GUSTO), also participated in the neuroimaging study. The GUSTO methodology has been published in detail [18]. The GUSTO cohort consisted of pregnant Asian women attending the first trimester antenatal ultrasound scan clinic at the National University Hospital (NUH) and KK Women’s and Children’s Hospital (KKH) in Singapore, which are the two major maternity hospitals in Singapore. Birth outcome and pregnancy measures were obtained from hospital records. The pregnant women and their partners were Singapore citizens or Permanent Residents of Chinese, Malay or Indian ethnic background. Socioeconomic status (household income) and prenatal exposure to alcohol (regular alcohol drinking) and tobacco (regular smoking, daily exposure to smoking at home and job) were extracted from survey questionnaires conducted as a part of a scheduled appointment during pregnancy. The GUSTO cohort study, including imaging procedures, was approved by the National Healthcare Group Domain Specific Review Board and the Sing Health Centralized Institutional Review Board. All clinical investigation has been conducted according to the principles expressed in the Declaration of Helsinki. Written consent was obtained from all guardians on behalf of the children enrolled in the study.

The current study only included neonates with diffusion tensor imaging (DTI), gestational age at birth greater or equal to 37 weeks, birth weight larger than 2500 g, and the last recorded APGAR≥9, to exclude effects of intra-uterine growth retardation or co-morbidities. Neonates of mothers with gestational diabetes, hypertension, and hypoglycemia were excluded from this study, as were neonates of mothers who reported consuming any alcohol during pregnancy.

### MRI Acquisition

Neonates underwent fast spin-echo T2-weighted MRI and single-shot echo-planar DTI scans using a 1.5-Tesla GE scanner at KKH’s Department of Diagnostic and Interventional Imaging between 5 and 17 days postpartum. The scans were acquired when subjects were sleeping in the scanner. No sedation was used and precautions were taken to reduce exposure to MRI scanner noise. A neonatologist was present during each scan. A pulse oximeter was used to monitor heart rate and oxygen saturation through out the entire scans.

The DTI imaging protocol is based on single-shot echo-planar DTI sequence (TR = 7000 ms; TE = 56 ms; flip angle = 90°, FOV = 200 mm×200 mm; matrix size = 64×64). 40 to 50 axial slices with 3.0 mm thickness were acquired parallel to the anterior–posterior commissure line. Nineteen diffusion weighted images (DWIs) with b = 600 sec/mm^2^ and 1 baseline with b = 0 sec/mm^2^ were obtained. All brain scans were reviewed by a neuroradiologist (M.V.F).

### DTI Analysis

Diffusion weighted images (DWIs) were first corrected within individual subjects for motion and eddy current distortions using affine transformation to the image without diffusion weighting. Six elements of the diffusion tensor were then determined, from which fractional anisotropy (FA) was calculated using multivariate least-square fitting.

We constructed a DTI atlas based on the unbiased diffeomorphic atlas generation algorithm [19] and detailed the processing procedure below. The FA image and the image without diffusion weighting of each subject were first aligned to those of the JHU neonate brain single-subject DTI atlas (resolution: 0.6×0.6×0.6 mm^3^, http://lbam.med.jhmi.edu/) [20] via affine and nonlinear large deformation diffeomorphic metric mapping (LDDMM) transformations [21]. These affine and nonlinear transformations were then applied to individual DWIs. The mean DWIs were obtained by averaging the DWIs with the corresponding gradient direction across all the subjects. The mean FA image was computed using the mean DWIs. These mean DWIs and FA were considered as a new atlas. This procedure was repeated three times until the intensity of DWIs was no longer changed. The mean DWIs and FA at the last iteration were defined as the final atlas and used as a common anatomical space.

DWIs of individual subjects were then aligned to the atlas based the same registration procedure as one used for the atlas generation. FA was obtained from the tensor calculation of DWIs in the atlas and used for the following voxel-based analysis.

### Statistical Analysis

Voxel-based analysis was performed to investigate the association between gestational age at birth and neonatal brain microstructure (FA) using SPM8. The FA images were smoothed with a Gaussian kernel with full width half maximum of 4 mm. Linear regression was performed conducted at every voxel in the white matter region. The regression model included gestational age at birth as the main factor and controlled for age at MRI. Most infants were scanned at the second week after birth. Gestational age was highly correlated with the postmenstrual age-at-scan (r = 0.966). As the aim of this study is to investigate the effects of gestational age at birth on the neonatal brain to assess effects of fetal maturity, we controlled for age at MRI (postmenstrual age-at-scan - gestational age at birth) in the regression analysis.

We also adjusted for birth weight [22], maternal age [22], monthly household income [22], [23], prenatal smoking exposure [22], [23], and ethnicity [24]–[26], on the basis of previous knowledge that these variables are potential confounders of younger gestational age. Family-wise error was computed for correcting multiple comparisons at a significance level of 0.001.

Birth weight was adjusted based on gestational age using linear regression with the mean-centered gestational age as a main factor on the GUSTO cohort. The residual was defined as adjusted birth weight that is statistically independent of gestational age. As small-for-gestational age has been identified as a risk factor for prematurity, we included birth weight as a covariate [22]. The brain grows rapidly in early life even in the first few weeks of life [27], [28] and hence it is crucial to justify the days from birth to the MRI visit. Several studies have suggested differences in brain morphology in different ethnic groups [24]–[26] and we thus also included ethnicity in the regression model. Also other reproductive risk factors have been identified, such as advanced maternal age and smoking, for which we adjusted in our analyses [22]. Furthermore, previous studies showed significant socioeconomic differences in preterm births, hence we have included household income [22].

## Results

### Demographics

Among 189 neonates who underwent MRI scans, 93 were included in the current study. We excluded 13 neonates with gestational age at birth less than 37 weeks, 11 with birth weight less than 2500 g, 2 with a 5-min Apgar score less than 9, and 52 with no DTI data. Moreover, our study also excluded infants whose mothers had gestational diabetes (n = 22), hypertension (n = 5), and hypoglycemia (n = 1) during pregnancy. Mothers who reported consuming any alcohol during pregnancy (n = 3) were also excluded from this study. Hence, the total sample involved in this study was 93. **Table 1** lists the demographic information of this study.

**Table 1:** Demographics.

		Sample (n = 93)
**Gestational age (37–41 weeks), mean (SD)**		38.8 (0.9)
**Age on the MRI day (days), mean (SD)**		9.9 (2.3)
**Birth Weight (gram), mean (SD)**		3115.6 (320)
**Adjusted Birth Weight (gram), mean (SD)**		3103.4 (328)
**Gender, Male/female**		44/49
Maternal Age (year), mean (SD)*		29.2 (5.4)
**Maternal Education Level, %**	**University**	14.0
	**GCE**	26.9
	**ITE_NTC**	15.1
	**Secondary**	37.6
	**Primary**	4.3
	**Others**	2.2
**Ethnicity, %**	**Chinese**	39.8
	**Malay**	44.1
	**Indian**	16.1
**Monthly Household** Income (S$), % *	**≤999**	4.3
	**1000∼1999**	18.3
	**2000∼3999**	40.9
	**4000∼5999**	22.6
	**≥6000**	14.0
**Prenatal Smoking Exposure, % yes**		52.7

Abbreviations. SD – standard deviation; ITE – Institute of Technical Education; NTC – National Technical Certificate; GCE – General Certificate of Education.

### Gestational Age at Birth and Fractional Anisotropy

**Figure 1:**
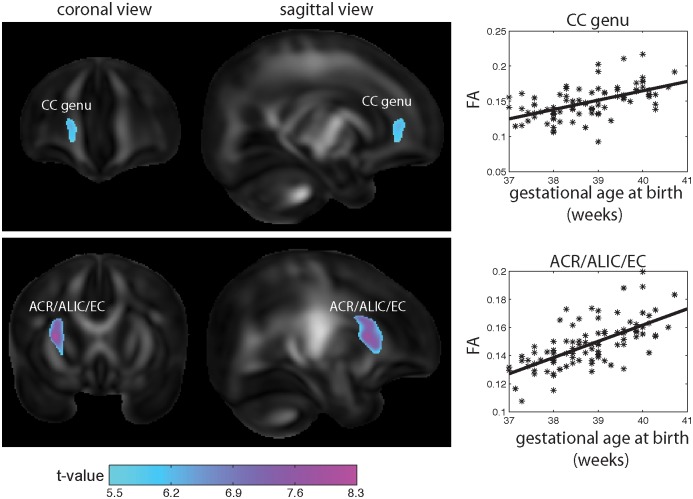
Association between gestational age at birth and fractional anisotropy of the neonatal brain. The left two columns show the statistical maps for the associations between gestational age at birth and fractional anisotropy of the neonatal brain, which were obtained from voxel-based analysis. The third column shows the scatter plots of FA in the CC region (top row) and in ACR/ALIC/EC regions (bottom row) highlighted in the left panels in relation with gestational age at birth. Abbreviations: CC --- corpus callosum; ACR --- anterior corona radiata; ALIC --- anterior limb of internal capsule; EC --- external capsule.

**Fig. 1** shows the statistical map for the association between gestational age at birth and FA of the neonatal brain. Adjusting for days from birth to MRI, adjusted birth weight, maternal age, monthly household income, prenatal smoking exposure, and ethnicity, we found that greater gestational age at birth was associated with larger FA values in two clusters. One cluster was located at the corpus callosum, specifically the genu (CC; corrected p<0.001). The mean FA in this region in relation with gestational age at birth is shown on the right panel of the first row in Fig. 1. The second cluster was at the right anterior corona radiata (ACR), anterior limb of internal capsule (ALIC), and external capsule (EC) (corrected p<0.001). The mean FA in this region in relation with gestational age at birth is shown on the right panel of the second row in **Fig. 1**. Our study did not reveal any non-linear relationship between brain measures and gestational age at birth.

We performed two additional analyses. First, we replaced gestational age at birth with post-conception age in the linear regression model. The effects of post-conception age on FA are the same as those of gestational age at birth. This is not surprising as the correlation of gestational age at birth and post-conception age at MRI is 0.966. Second, we examined the growth pattern of FA after birth, that is, the growth pattern of FA as a function of age at MRI, where age at MRI is defined as the difference between post-conception age and gestational age at birth. No significant statistical results were found after correcting for multiple comparisons. Hence, this suggests that the findings shown in **Fig. 1** are mainly due to gestational age at birth.

## Discussion

Our cross-sectional findings show an association between gestational age at birth within the normal range and brain maturation measured with diffusion tensor imaging, shortly after birth. Specifically, younger gestational age at birth within the “term” window associates with reduced white matter organization in the CC, ACR, ALIC, and EC in comparison to older gestational age within the “term” window. These brain regions are parts of the cortical-striatal-thalamic neural circuit. This circuit is functionally important as is has been related to sensory functions, executive functions, and working memory [29]–[31].

Despite the fact that tracts, such as the CC, do not fully mature until adulthood, the majority, including the internal capsule (IC) with its extensions to the thalamus and basal ganglia, are in the limited myelinated brain regions at birth [32]. These tracts may be vulnerable to brain damage in the perinatal and early postnatal period [33], [34], and are involved in several neural circuits supporting basic brain functions at birth. For example, the ALIC is a major cortico-subcortical white matter bundle that contains fibers running from the thalamus to the basal ganglia as well as connecting the thalamus to the frontal lobe. Coordinated activity of the thalamus, basal ganglia and frontal lobes is essential for regulating sensori-motor functioning. Likewise, the corona radiata is the continuation of the IC as it makes its way to sensorimotor cortex in and near the central sulcus [35].

During the second trimester of gestation, the commissural fibers [36] and projection fibers (ALIC and CR) are visible using tractography in *ex-vivo* DTI [1], [37]. It has been recently found that structures like ACR, ALIC and EC are likely to develop rapidly at near-term age during the third trimester, when growth and neurogenesis of the fetal brain increase [38]. Indeed, our findings show variation of *in utero* brain maturation in these early developing structures in healthy term infants. This is consistent with previously reported associations in studies with premature infants and as such implies a consistent pattern of white matter development in the fetal brain. Those studies indeed found an association between gestational age at birth and maturation of the posterior limb of the internal capsule (PLIC) [39], [40], [41], [42]. Also, associations between gestational age and ALIC have been found before [38], [40].

And one previous study, although performed in very-low-birth-weight and premature children, also reported higher FA in the posterior regions within the CR. The same study found a positive association between gestational age at birth and the EC for Mean Diffusivity but not FA [38]. The authors suggested a slower growth of this region in infants with higher gestational age, possibly caused by accelerated growth in these regions upon birth in preterm neonates [38]. However, our study represents the observance of associations between gestational age at birth and higher FA in CR and EC in term born infants. The formation of the CC is relatively more advanced in the frontal lobe than other brain regions during the fetal stage, which may suggest the importance of inter-hemispherical communication in the frontal region beginning early in life [43].

Previous work examining very preterm and near term infants has also documented an association between CC development and gestational age [38], [44]–[46].

Indeed, most of this research demonstrates that FA and diffusion variations in the CC correlate significantly with gestational age [36], [38], [47], although some found an association with birth weight instead of gestational age [48]. Nevertheless, extreme prematurity and extremely low birth weights imply multiple complications that could influence brain development and may not be applicable to healthy term infants. Indeed, Bonfacio (2010) did a first MRI scan (of a serially DTI scans) as soon as the premature infants born between 24 and 33 weeks of gestation were deemed clinically stable by the attending neonatologist, in between 30 weeks and 33 weeks of postmenstrual age. They found that brain microstructure was independent of (extremely) premature birth, and that only brain injury and co-morbid conditions were important determinant of microstructure maturation [49]. This suggests that white matter abnormalities found with brain DTI in clinical samples may be due to the overwhelming influences of brain injury and does not inform us about the influence of gestational age on brain maturation in the “term window” of healthy infants.

Other studies about CC compared preterm and term children with neuroimaging, but the timings of scanning were after a significant postnatal period [3], [39], [40], [50]–[52]. These studies consistently report reduced CC in premature children [3], [39], [50], [51] in comparison to nearly preterm or term born infants [3], [40]. However, the multiple confounders that influence postnatal brain maturation in the time between birth and imaging comprise the ability to directly associate gestational age at birth to *in utero* brain development.

Although other studies also found associations between gestational age and the thalamus in premature infants, we did not find this in our study. This can be explained by previous findings of myelination of the thalamus around the second trimester, and makes this structure more vulnerable for effects of prematurity instead of effects of gestational age within the “term window” [38], [40].

Although some studies in preterm infants suggest that differences in brain trajectories at birth can be persistent over time [50], [53], other studies in preterm infants suggest that early life brain development is ongoing and independent of the degree of gestational age [54]–[56]. These latter studies suggest that many post birth environmental factors can potentially influence brain functioning, which suggests plasticity of the brain. It is still unclear if variation of *in utero* brain maturation in healthy term infants will also have implications for functional outcomes. It is known that there is rapid rate of growth of the brain, inclusive subcortical areas, during the first 3 months after birth [13]. On the other hand a previous study in 6 years old Singaporean boys demonstrated an association between gestational age at birth between 37 and 41 weeks and reaction time on a stop-signal test that assesses executive functioning dependent upon fronto-striatal functioning [57], which suggest that gestational age within the “term” window may have longer lasting effects on functioning. Future studies are required to investigate the functional significance of the variability in brain maturation found with DTI in healthy infants at birth and its predictive validity.

A major strength of this study is the use of DTI techniques to image newborn infants soon after birth. Our study focused on a healthy sample, excluding infants born preterm as well as infants with very low birth weights, low Apgar scores and complications during pregnancy. These strict rules for subject inclusion allowed us to examine the effects of gestational age without the associated influences of pathologies common in pre-term and high-risk pregnancies. Although inclusion of infants with APGAR score ≥9 after maximum of 10 minutes may be over-conservative, we only excluded two infants based on an APGAR<9, which will not have changed our results.

Still, our study has limitations. First, FA indices should be interpreted with caution without knowledge of the possible effects of water concentration in the newborn infant brain [39]. We employed FA to indicate the integrity of the white matter in early life. Relatively greater FA in regions such as ALIC and CC at birth may indicate greater coherence in fibers organization and greater proliferation of oligodendrocytes prior to myelin ensheathment [58]. Second, as we did scan the infants as soon as possible after birth, we were not able to compare them at equivalent gestational ages. Hence, we do not know how the brain regions of the infants that were born with a relatively younger gestational age will evolve, and if these infants will eventually “catch up” in brain maturation over time. There was still a postnatal period of brain maturation of maximum 2.5 weeks. As this is a significant period given the fast brain development in the early postnatal period [13] we did not only adjust for days after birth but also have run additional analyses using post-conception age instead of age at birth to explore the general maturation due to age since conception.

Third, because DTI is still a relatively new technique, the functional significance of these findings is unclear. Our study did not explore the functional outcomes of the differences in brain maturation in term born infants. However, the GUSTO cohort will be followed over the coming years, which will help us to explore not only if some variation in brain maturation will persist at later ages but also the functional significance of our findings of early variation of *in utero* brain development, such as effects on executive functioning.

## Conclusions

We report that a relatively higher gestational age at birth in healthy term infants is associated with more white matter maturity in the CC, as well as in ALIC, ACR and EC, in comparison to healthy term infants born with a relatively lower gestational age. Future studies in term infants are needed to explore the effects of variations in brain development at birth and later brain development and functional outcomes.
